# Feasibility, acceptability, and preliminary efficacy of a single-arm, remotely-delivered health coaching intervention to increase physical activity and reduce sedentary behavior during pregnancy

**DOI:** 10.1186/s12884-022-05073-4

**Published:** 2022-10-02

**Authors:** Kara M. Whitaker, Melissa A. Jones, Jaclyn Dziewior, Megan Anderson, Chelsie Anderson, Bethany Barone Gibbs, Lucas J. Carr

**Affiliations:** 1 Department of Health and Human Physiology, University of Iowa, 225 S. Grand Ave, Iowa City, IA 52242 USA; 2 Department of Epidemiology, University of Iowa, Iowa City, USA; 3 Department of Epidemiology and Biostatistics, West Virginia University, Morgantown, USA

**Keywords:** Accelerometry, Exercise, Gestation, Health behavior

## Abstract

**Background:**

Interventions targeting physical activity and sedentary behavior concurrently in pregnancy may be an ideal strategy to reduce the risk of pregnancy complications. We assessed the feasibility, acceptability, and preliminary efficacy of a single-arm, remotely-delivered health coaching intervention to promote physical activity and reduce sedentary behavior in pregnancy.

**Methods:**

Women (*n* = 34) between 8 and 12 weeks gestation were recruited to take part in the INcreasing Steps in PREgnancy (INSPiRE) study. Participants were given an activity tracker (Fitbit Inspire) and met virtually with their health coach throughout the second and third trimesters of pregnancy. Feasibility was based on enrollment, retention, and adherence rates. Acceptance was assessed using a process evaluation survey. Intervention efficacy was based on activPAL data obtained at baseline and the end of the second trimester.

**Results:**

Feasibility objectives were met, with greater than 70% enrollment, 97% retention, and 99% adherence. All participants reported high levels of satisfaction with the program. ActivPAL data indicated statistically significant increases in daily steps (+ 1715.8 steps/day, Cohen’s *d* = 0.97), stepping time (+ 1.9%, *d* = 0.75), standing time (+ 2.3%, *d* = 0.29), and decreases in total sedentary time (− 4.2%, *d* = 0.43) and sedentary bouts of 30 minutes (− 4.1%, *d* = 0.36) from baseline to the end of the second trimester, all *p* < 0.05. Decreases were also observed in sedentary bouts of 60 minutes (− 3.9%, *d* = 0.40), but this was not statistically significant.

**Conclusions:**

The INSPiRE study demonstrated feasibility, high acceptability, and preliminary efficacy for improving movement behaviors in women during pregnancy, supporting future testing in a randomized controlled trial.

## Background

Physical activity during pregnancy is safe for the mother and baby and has many health benefits, including a lower risk of maternal gestational hypertension, preeclampsia, and gestational diabetes [1, 2]. However, pregnant women are less likely to meet the recommended guidelines of 150 minutes per week of moderate-intensity physical activity compared to non-pregnant women (15.9% vs. 26.1%, respectively) [3]. Pregnant women experience typical but also unique barriers to moderate-to-vigorous intensity physical activity (MVPA), including misperceptions about the risks of physical activity when pregnant, fatigue, and nausea [4, 5]. Evidence also indicates that high sedentary behavior, defined as a seated/reclining posture and low-intensity activity (≤1.5 x basal metabolic rate) [6], may have adverse consequences during pregnancy. Data from our research group indicates that high sedentary behavior during pregnancy is associated with an increased risk of hypertensive disorders in pregnancy and other adverse pregnancy outcomes, independent of physical activity level [7]. Similarly, high sedentary behavior has also been identified as a risk factor for earlier gestational age at delivery and inhibited fetal growth [8]. Thus, encouraging physical activity participation while concurrently promoting less sedentary behavior during pregnancy to reduce the risk of pregnancy complications may be an ideal strategy.

In the last decade, there has been an increase in behavioral interventions to improve MVPA during pregnancy, with mixed evidence on the efficacy for increasing physical activity [9–11]. However, most of these interventions were delivered in clinical settings and required women to attend in-person exercise sessions, thus increasing participant burden and reducing adherence [9, 10]. Few, if any, studies in pregnant women have attempted to reduce sedentary behavior. This gap in research has important public health implications as replacing sedentary time with lower-intensity physical activities may be a more attainable goal for pregnant women, as compared to structured MVPA programs, and may still result in health benefits. Research by Davenport and colleagues, including the 2019 Canadian guidelines for physical activity throughout pregnancy [12], shows all improvements in physical activity, even lower levels that do not meet the guidelines, can contribute to clinically meaningful health benefits in pregnancy. In addition, remotely delivered interventions, or programs that do not require in-person contact and include patient monitoring devices (e.g. Fitbit), are growing in popularity due to their potential to reach large numbers of individuals, low participant burden, and additional benefit of limiting exposure during the COVID-19 pandemic [13]. These types of interventions have shown to be effective at increasing physical activity levels in non-pregnant populations [14]. However, the feasibility, acceptability, and efficacy of a remotely-delivered intervention designed to increase physical activity and reduce sedentary behavior in pregnancy is unknown.

To address these gaps, the purpose of this pilot study was to test the feasibility, acceptability, and preliminary efficacy of a single-arm virtual health coaching intervention on physical activity and sedentary behavior during pregnancy among insufficiently active women. We hypothesized that participants would increase daily steps and decrease total sedentary time over the course of the intensive intervention phase (second trimester). Further, we hypothesized that participants would maintain or have minimal decreases in daily steps from the end of the intensive intervention phase through the follow-up phase (third trimester). We also obtained information from electronic medical records on pregnancy complications and infant outcomes as exploratory outcomes to inform future research.

## Materials and methods

### Study overview

The INcreasing Steps in PREgnancy (INSPiRE) study was a single-arm, remotely-delivered health coaching pilot intervention trial to assess the feasibility, acceptability, and preliminary efficacy of a physical activity and sedentary behavior intervention in pregnancy that took place from July 2020-August 2021 in Iowa City, Iowa, United States. The overall goals of the intervention were to increase daily steps and to reduce sedentary time across the second trimester of pregnancy (intensive intervention phase) and to maintain activity levels based on comfort level across the third trimester of pregnancy (follow-up phase). Health coaching sessions were weekly for the first month (initial weekly session beginning at 10-14 weeks gestation), bi-monthly in months 2-4 (initial bi-monthly session beginning at 15-19 weeks gestation), and monthly until delivery (initial monthly session beginning at 27-31 weeks gestation), for a total of 12 contacts. Participants also received one text message between coaching contacts to reinforce content discussed in coaching sessions. As seen in the sample intervention contact time (Table 1), physical activity and sedentary time were measured with an activPAL3 micro for 7 days in the first trimester (8-12 weeks gestation, ~ 2 weeks prior to the first health coaching session), and again at the end of the intensive intervention phase. Questionnaires were administered after activPAL wear in the first trimester but prior to beginning the health coaching sessions, and again at the end of the intensive intervention phase. The Fitbit monitor was mailed or delivered to the participant 1-3 days prior to their first health coaching session. Participant satisfaction questionnaires were administered after conclusion of the program.

**Table 1:** Sample Intervention Contact Timeline

**1st Trimester (8-12 Weeks Gestation)**													
8		9			10 Screen/Consent			11 ■			12 ♦		
**2nd Trimester (13-26 Weeks Gestation) – Intensive Intervention Phase**													
13 ●	14 ●	15 ●	16 ●	17	18 ●	19	20 ●	21	22 ●	23	24 ●	25	26 ■ ♦ ●
**3rd Trimester (27-40 Weeks Gestation) – Follow-up Phase**													
27	28	29	30 ●	31	32	33	34 ●	35	36	37	38 ● ♦	39	40

■ = activPAL wear

♦ = Questionnaires administered

● = Health coaching session

The University of Iowa Institutional Review Board approved all research procedures, and all participants provided written informed consent. This study was retrospectively registered at ClinicalTrials.gov on 13/07/2022 (NCT05455008). The datasets used and analyzed for this study are available in the University of Iowa Institutional Repository [15].

### Participants

Participants were recruited to complete an electronic screening form using a university-wide mass e-mail campaign sent to all University of Iowa employees and students. A secondary recruitment strategy included sending targeted emails to new prenatal patients who enrolled in the Maternal Fetal Tissue Bank of the Women’s Health Tissue Repository at the University of Iowa [16] and consented to be contacted for other research studies. Using these methods, we recruited a convenience sample of women who were less than 13 weeks pregnant, between 18 and 44 years of age, owned a smart phone, were able to speak, comprehend, read, and write in English, and self-reported insufficient activity as determined by the PARmed-X for pregnancy (exercising less than 150 minutes per week) and/or less than 7000 steps/day. Women were excluded for the following reasons: currently enrolled in another research study about exercise, physical limitations that prevented exercise, instructed by a physician to not exercise during pregnancy, hospitalized for a psychiatric disorder in the past 6 months, absolute or relative contraindication to exercise as determined by the PARmed-X for pregnancy, or other serious medical conditions. We originally planned to exclude participants after consent if they averaged > 7000 steps/day at baseline, as determined by the activPAL. However, many women were achieving more than 7000 steps/day; thus, the exclusion criteria were adjusted to > 9000 steps/day to accommodate more participants.

### Intervention description

The INSPiRE intervention included: (1) ~ 12 structured one-on-one virtual health coaching sessions delivered via Zoom or telephone, (2) a Fitbit Inspire wrist monitor to encourage physical activity self-monitoring, (3) a mobile app (Healthie) to share educational materials and facilitate communication between the health coach and participant, including real time data collection of Fitbit information, and (4) printed tip sheets that were mailed to participants to reinforce content discussed during coaching sessions. Each health coaching session emphasized a specific topic area, as outlined in Table 2. All coaching sessions also included a discussion of Fitbit-determined steps, if goals from the prior session were met, and goal setting for the subsequent session. The first coaching session was approximately 30-45 minutes in length, remaining coaching sessions ranged from 15 to 30 minutes in length.

**Table 2:** Brief overview of study contacts

Session	Frequency	Overview of content
1	Weekly	Physical activity benefits and safety
2	Weekly	Physical activity tips and resources
3	Weekly	Targeting motivation
4	Weekly	Making time for physical activity
5	Bi-monthly	Social Support
6	Bi-monthly	Self-Efficacy
7	Bi-monthly	Making physical activity a habit
8	Bi-monthly	Long-term maintenance
9	Bi-monthly	Barriers to physical activity
10	Monthly	Pregnancy exercise maintenance
11	Monthly	Pregnancy exercise maintenance & maintaining exercise after baby
12	Monthly	Pregnancy exercise maintenance

All contacts by Zoom or telephone

The primary goal of the program was to increase steps by approximately 10% between each coaching session until reaching up to 10,000 steps/day, pending participant comfort and health status. This target step goal is consistent with other physical activity interventions delivered during pregnancy [17]. Participants were encouraged to choose physical activities that they enjoyed. The secondary goal of the program was to decrease total sedentary time by incorporating short active breaks throughout the day. Participants worked with their health coach to set goals for steps and sedentary behavior.

Prior to the first coaching session, the participant received a Fitbit Inspire, Fitbit use and care instructions, and 12 printed motivational tip-sheets that corresponded to each health coaching session. In addition, participants were instructed to download two applications on their mobile device to facilitate self-monitoring and communication between the health coach and participant (Fitbit app and Healthie app).

The health coaching sessions were adapted with permission from the Healthy Mom study [18, 19], a telephone-based exercise intervention for the prevention of postpartum depression. The intervention sessions were grounded in Self Determination Theory [20, 21] and Social Cognitive Theory [22]. The two health coaches had bachelor’s degrees in health-related fields and were trained to use motivational interviewing strategies [23]. At the first health coaching session, the health coach reviewed the participant’s data from the activPAL worn during the first trimester of pregnancy to review baseline steps and sedentary time and identify opportunities for improvement. Exercise safety was discussed according to guidelines recommended by the American College of Obstetrics and Gynecology (ACOG), including relative and absolute contraindications to exercise [24]. The health coach also verified that the participant had downloaded the apps required for the program and that all data was syncing appropriately; assistance was provided if the apps had not yet been downloaded or if any problems were identified.

### Measures

Baseline questionnaires were administered using REDCap following activPAL wear but prior to the initial health coaching session. Demographic information included: age, race/ethnicity, education, marital status, health insurance coverage, annual household income, parity, and smoking status. Further, because this intervention took place from 2020 to 2021 during the COVID-19 pandemic, we also asked if activity levels had changed due to the COVID-19 pandemic, if source of income was lost due to the COVID-19 pandemic, and whether the COVID-19 pandemic affected their regular childcare (if participants reported having ≥1 dependents living at home) as these factors could influence activity and sedentary behaviors. Medical and reproductive history were also assessed, including prior pregnancy complications (if reporting ≥1 prior pregnancy), pre-pregnancy weight, and use of infertility treatments for the current pregnancy. At the end of the intensive intervention phase, changes to demographics or medical and reproductive history were captured. At the end of the follow-up phase, program acceptability was assessed. After delivery, medical data was collected from participant’s medical records, including the following pregnancy complications: pregnancy hypertension, preeclampsia/eclampsia, gestational diabetes, intrauterine growth restriction, and preterm delivery. Labor and delivery data abstracted included weight at delivery (used to calculate total gestational weight gain), and mode of delivery (c-section or vaginal). Infant outcomes included child sex, weight, length, head circumference, and Apgar scores at birth. Child sex, weight, and length were used to calculate BMI Z-scores [25].

To examine the acceptability of the intervention, a program evaluation survey was administered at the conclusion of the follow-up phase. Participants were asked how satisfied they were with the program overall, with response options including very satisfied, satisfied, neutral, dissatisfied, and very dissatisfied. A 5-point Likert scale (1 = strongly agree, 5 = strongly disagree) was used to assess how well participants liked various components of the intervention (health coaching sessions, Fitbit, Healthie, tip sheets, and activPAL). Questions also assessed participants’ perceived changes in physical activity and quality of life since beginning the program. Open-ended questions gave participants an opportunity to provide specific feedback on what aspects they liked and disliked and how the program could be improved.

Physical activity and sedentary behavior were measured objectively for 7 days using the activPAL3 micro at baseline (prior to the initial health coaching session) and again at the end of the intensive intervention phase. Participants were mailed the monitor with instructions and a monitor wear log to denote sleep and non-wear periods and were asked to return the monitor and log in a pre-paid envelopment. Participants were instructed to wear the activPAL3 on the anterior thigh using the provided waterproof, transparent dressing for 24 hours per day, including while bathing, with removal only when swimming to prevent monitor loss. Event data were exported using PAL Technologies software (version 8; PAL Technologies, Glasglow, Scotland); nonwear and sleep times were removed using participant diaries. To assess preliminary efficacy, our primary outcome was change in steps per day for physical activity and change in total sedentary min/day for sedentary time. Additional measures included change in stepping min/day, standing min/day, and sedentary min/day accumulated in bouts of 30 and 60 minutes. Total wear time and total wear days were also captured; data were considered valid with ≥5 days of wear with ≥10 hours of wear time each day [26, 27].

A secondary approach to assess physical activity was change in steps per day measured with the wrist-worn Fitbit INSPiRE, which was worn throughout the duration of the intervention. Fitbit devices have shown high levels of reliability and validity for step counts compared to direct observation and the ActiGraph accelerometer [28, 29]. Step count data was automatically transferred from Fitbit to Healthie when participants synced their Fitbit device. If data transfer from Fitbit to Healthie did not occur for three consecutive days, participants were reminded by text message to sync their Fitbit device by their health coach. We assessed change in steps per day (averaged between health coaching sessions) using the Fitbit device from baseline to the end of the intensive intervention phase, as well as change from the end of the intensive intervention phase through the final health coaching session. As reported in other populations [30, 31], data were considered valid if step counts were ≥ 1000 steps per day; values below this threshold likely represent partial wear days.

### Statistical analysis

We sought to have 80% power to detect a change of 1000 steps per day with an alpha of 0.05. An effect size of 0.53 was calculated using an estimated average change of 1000 ± 1900 steps/day, based on data reported from another physical activity intervention during pregnancy [32]. Using these criteria it was estimated that 31 women were needed. To account for miscarriages after consent, drop-out, and loss to follow-up (estimated rate of 15%), we recruited an additional five women, for a total of 36 participants.

Feasibility was defined by three components: (1) recruitment and enrollment of at least 50% of eligible women who completed a screening form, (2) retention of 85% (~ 31/36) or more participants from baseline through delivery (defined as completing at least half of health coaching sessions and medical record abstraction), and (3) adherence if the participants attended 75% or more of the health coaching sessions on average and wore and synced their Fitbit device on at least 75% of days from baseline through the end of the intervention.

Acceptability was defined as at least 75% of the respondents indicating they were satisfied or very satisfied with the program overall.

Preliminary efficacy was determined using paired t-tests to assess activPAL-measured changes in physical activity measures (steps/day, stepping min/day, standing min/day) and sedentary behavior measures (sedentary min/day, total and in bouts of 30 and 60 minutes) from baseline through the end of the intensive intervention phase. Due to significant differences in total waking wear time between assessments, stepping, standing, and sedentary min are reported as a percentage of total waking wear time. Cohen’s *d* or the standardized mean difference was calculated to estimate the effect size of the observed change in our primary and secondary measures, with effect sizes of 0.2, 0.5, and 0.8 considered small, medium, and large, respectively [33].

As a secondary outcome we examined changes in Fitbit-measured steps/day averaged between each health coaching session (e.g., daily steps between sessions 1 and 2 were averaged to represent session 1 steps). Linear mixed models examined average changes in steps between study sessions from study baseline (session 1) to the end of the intensive intervention phase (session 9), and separately from the end of the intensive intervention phase through the end of the program (session 12). Intervention session was the independent variable and average steps/day between intervention sessions were the dependent variables for analysis [31]. SAS v9.4 was used for all analyses.

## Results

### Recruitment

**Fig. 1:**
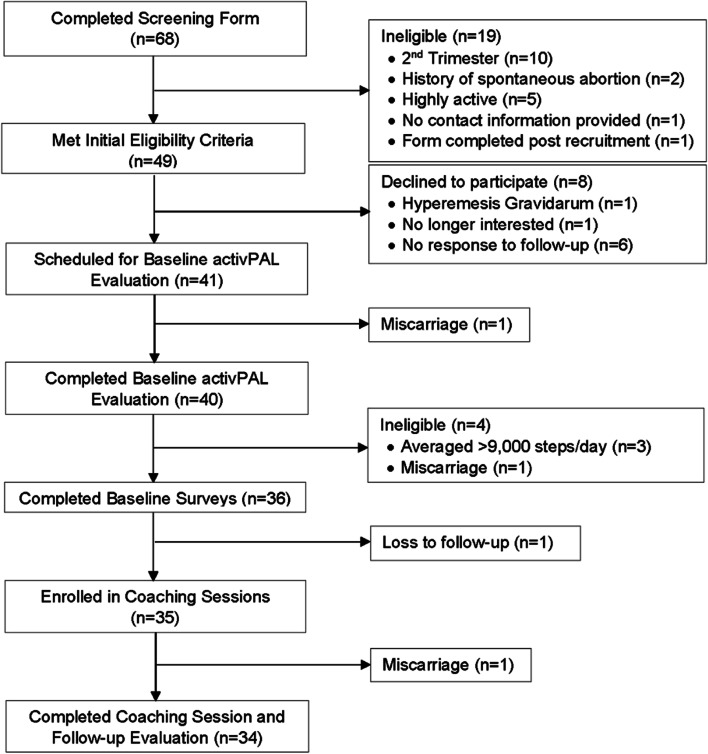
Participant flow diagram

The flow of participant recruitment is depicted in Fig. 1. A total of 68 potential participants completed the screening form, of which 49 met initial eligibility criteria. The most common reasons for exclusion were late gestational age (≥13 weeks gestation) and achieving more than 150 minutes per week of MVPA and/or more than 7000 steps/day via self-report. A total of 41/49 who met initial eligibility criteria were scheduled for baseline activPAL evaluation. One participant experienced a miscarriage while completing the baseline activPAL wear protocol. After wearing the activPAL monitor, 4/40 were no longer eligible because they averaged > 9000 steps/day or experienced a miscarriage. Of the 36 participants who completed baseline surveys, 35 began health coaching sessions and 34 completed the full program. Those who were deemed ineligible after completing baseline surveys (*n* = 2) appeared to be younger, lower income, and had higher pre-pregnancy BMI than those included in the study sample. Recruitment ended after reaching the target enrollment number.

### Participants

As seen in Table 3, the average age of participants who completed the program was 31.1 ± 3.3 years (range 25.0-39.0 years). The majority were non-Hispanic White, highly educated, married, and had private health insurance. Nearly 80% of women reported that the COVID-19 pandemic had decreased their physical activity, with approximately 15% reporting additional childcare responsibilities due to the pandemic. Few women reported a history of pregnancy complications, and more than 50% had a normal pre-pregnancy BMI.

**Table 3:** Participant characteristics from the INSPiRE Study (*N* = 34)

Participant Characteristics	
Age, mean years ± SD	31.1 ± 3.3
Race/ethnicity, n(%)	
Non-Hispanic White	31 (91.2)
Non-Hispanic Black	1 (2.9)
Non-Hispanic Asian	1 (2.9)
Hispanic White	1 (2.9)
Education, n(%)	
Some College or Associates Degree	1 (2.9)
Bachelor’s Degree	13 (38.2)
Graduate Degree	20 (58.8)
Married	34 (100.0)
Private health insurance	34 (100.0)
Annual family household income	
< $49,000	3 (8.9)
$50,000-$99,000	12 (35.3)
$100,000-$149,000	13 (38.2)
≥ $150,000	6 (17.6)
Parity, n(%)	
Primipara	17 (50.0)
Multipara	17 (50.0)
Current smoker, n(%)	0 (0.0)
Changes in physical activity due to the COVID-19 pandemic	
Physical activity decreased	27 (79.4)
No change	6 (17.7)
Physical activity increased	1 (2.9)
Source of income lost due to the COVID-19 pandemic	1 (2.9)
Changes in childcare due to the COVID-19 pandemic^a^	
Difficulty arranging for childcare	3 (8.8)
Paid more for childcare	1 (2.9)
Spouse/partner or I cared for our children ourselves	5 (14.7)
My regular childcare was not affected	6 (17.7)
I do have have a child in childcare	14 (44.1)
Pregnancy History, n(%), *n* = 17	
History of Gestational Hypertension	2 (11.8)
History of Preeclampsia	2 (11.8)
History of Gestational Diabetes	2 (11.8)
History of Preterm birth	0 (0.0)
Prepregnancy BMI, mean kg/m^2^ ± SD	26.4 ± 6.1
Prepregnancy BMI category, n(%)	
Normal (18.5-24.9 kg/m^2^)	18 (52.9)
Overweight (25.0-29.9 kg/m^2^)	6 (17.7)
Obese (≥30.0 kg/m^2^)	10 (29.4)
Infertility treatment, n(%)	5 (14.7)

^a^More than one response option was possible

Pregnancy outcomes are presented in Table 4. Average gestational age at delivery was 38.8 ± 1.3 weeks, with only one participant delivering preterm. A total of 8 women or 23.5% of the study sample had at least one adverse pregnancy outcome. Approximately 50% of women experienced weight gain that exceeded the Institute of Medicine gestational weight gain guidelines [34]. Infants on average weighed 3387 g or 7.5 pounds at birth and had high Apgar scores (range 7-9).

**Table 4:** Pregnancy outcomes from the INSPiRE Study (*N* = 34)

Pregnancy Outcomes	
Gestational age at delivery, mean weeks ± SD	38.8 ± 1.3
Adverse Pregnancy Outcomes	
Gestational hypertension, n(%)	3 (8.8)
Preeclampsia, n(%)	0 (0.0)
Gestational diabetes, n(%)	2 (5.9)
Preterm delivery, n(%)	1 (2.9)
Intrauterine growth restriction, n(%)	3 (8.8)
All adverse pregnancy outcomes, n(%)	8 (23.5)
Gestational weight gain, mean kg ± SD (*n* = 29)	13.3 ± 5.8
Institute of Medicine gestational weight gain guidelines, n(%) (n = 29)	
Below	3 (10.4)
Within	12 (41.4)
Above	14 (48.3)
Mode of delivery	
Vaginal delivery	30 (88.2)
Cesarean delivery	4 (11.8)
Infant outcomes	
Male, n(%)	17 (51.2)
Birthweight, mean grams ± SD	3386.6 ± 544.3
Birth length, mean cm ± SD (*n* = 29)	50.8 ± 3.0
BMI Z-Score, mean ± SD (*n* = 29)	−0.33 ± 1.2
1-minute Apgar Scores, mean ± SD (*n* = 32)	7.8 ± 1.0
5-minute Apgar Scores, mean ± SD (*n* = 32)	8.9 ± 0.3

### Feasibility

To determine feasibility, the first objective was to recruit and enroll at least 50% of eligible women who completed a screening form. As seen in Fig. 1, a total of 49 women who completed the study screening form were deemed eligible, of which 41 consented to participate and 35 enrolled in the coaching program (71% recruitment and enrollment). Our second objective was to retain 85% or more participants from baseline to delivery. A total of 34/35 of the women who enrolled completed the program (97% retention). The one participant who did not complete the program had an early second trimester miscarriage. The third objective was to assess adherence through attendance at 75% or more of health coaching sessions and syncing of their Fitbit device on 75% or more days. Of the 288 possible health coaching sessions (12 sessions × 34 participants), a total of 286 sessions were held (99% adherence). The two sessions that were not held (both session 12) were due to birth of the baby prior to the final health coaching session. Step data were missing or deemed invalid (< 1000 steps/day) on 81 of 5913 possible days (98.6% wear and syncing compliance), with most missing days occurring between session 11 and 12. All participants had excellent adherence, syncing their Fitbit device on 95% or more of possible wear days.

### Acceptability

Of the 34 participants who completed the program, 32 completed the program evaluation survey (94%). All participants who completed the program evaluation indicated they were very satisfied (*n* = 24, 75%) or satisfied (*n* = 8, 25%) with the program and would recommend the program to other pregnant women (*n* = 32, 100%).

Most women strongly agreed or agreed that the health coaching sessions helped them increase their physical activity (*n* = 31, 97%) and decrease the time they spent sitting during the day (*n* = 30, 94%). All participants agreed that they felt supported by their health coach, their questions were answered, and that it was easy to talk to their health coach (*n* = 32, 100%). Nearly all participants strongly agreed or agreed that they enjoyed having the health coaching sessions remotely (*n* = 31, 97%). Feedback also indicated that the duration and number of sessions was appropriate with only one participant indicating a desire for more sessions (3%) and two indicating a desire for fewer sessions (6%).

Feedback was also collected concerning the Fitbit, Healthie app, tip sheets, and activPAL used during the intervention. All individuals agreed that their Fitbit was easy to use and helped them achieve their goals (*n* = 32, 100%), with most indicating that it was comfortable (*n* = 28, 88%) and easy to remember to wear (*n* = 25, 78%) and charge (*n* = 24, 75%). The Healthie app, however, was not used regularly by most participants (*n* = 30, 94%). Although some participants indicated they referenced the tip sheets during their pregnancy (*n* = 16, 50%), most did not agree that they helped increase their physical activity (*n* = 20, 63%). Most participants stated that the activPAL device was comfortable to wear (*n* = 28, 88%).

The intervention appeared to also have a positive influence on participant’s quality of life. Most participants strongly agreed or agreed that they felt healthier (*n* = 27, 84%) had more energy during the day (*n* = 23, 72%), and noticed an improvement in their mood at the conclusion of the study (*n* = 23, 72%).

Open-ended responses also assessed what aspects of the program participants liked and disliked and how the program could be improved. Participants most consistently stated that their favorite components of the program were setting and achieving goals (*n* = 10, 31%), social support provided by the health coaches (*n* = 8, 25%), and accountability (*n* = 8, 25%). Only 25 participants responded to the open-ended question assessing factors they disliked about the program, of which 7 (28%) stated there were no components they disliked. The most cited dislikes included having to wear the Fitbit or activPAL (*n* = 6, 24%) and wanting to focus on additional goals beyond physical activity and sedentary behavior, such as goals around sleep (*n* = 3, 12%). Of the 25 participants who responded to the question asking for suggestions for improvements, the majority indicated they had no suggestions for improvements (*n* = 16, 64%). However, two participants said they would have preferred at least one in-person meeting (8%), five participants expressed interest in discussing diet and nutrition information with their health coach (20%), and two indicated a desire to receive information on postpartum exercise (8%).

### Preliminary efficacy

For our primary outcome, we examined activPAL-measured changes in physical activity and sedentary behavior from before the start of the intervention (baseline) through the end of the intensive intervention phase (session 9). As seen in Table 5, all physical activity measures significantly increased from baseline to the end of the intensive intervention phase. Participants increased their daily steps (+ 1715.8 steps/day), stepping time (+ 1.9%; + 16.8 min/day), and standing time (2.3%; + 20.3 min/day). Calculated effect sizes were large for daily steps (0.97), medium-large for stepping time (0.75), and small for standing time (0.28). Women also had significant decreases in total sedentary time (− 4.2%; − 37.0 min/day) and time spent in sedentary bouts of at least 30 minutes (− 4.1%; − 36.1 min/day), *p* < 0.03. A reduction in sedentary bouts of at least 60 minutes was observed, but this was not statistically significant (− 3.9%; − 34.4 min/day). Calculated effect sizes were small-medium across sedentary measures (range 0.36-0.43). Total waking wear time was significantly different between assessments (+ 24.7 min/day).

**Table 5:** Changes in activPAL physical activity and sedentary time among INSPiRE participants from baseline to follow-up (*N* = 34)

activPAL assessments	Baseline 8-12 Weeks Gestation	End of Intensive Intervention 22-28 Weeks Gestation	Change from Baseline to End of Intensive Intervention	Cohen’s ***d***	***P***-value
**Physical activity**					
Steps/day	5654.3 ± 1823.3	7370.1 ± 1708.9	1715.8 ± 1770.7	0.971	< 0.001
Stepping, %	8.7 ± 2.8	10.6 ± 2.3	1.9 ± 2.3	0.746	< 0.001
Standing, %	23.4 ± 8.9	25.8 ± 7.1	2.3 ± 6.3	0.288	0.041
**Sedentary time**					
Total, %	67.8 ± 10.9	63.6 ± 8.4	−4.2 ± 8.0	0.434	0.004
30 min bouts, %	38.6 ± 13.1	34.5 ± 9.8	−4.1 ± 10.3	0.356	0.026
60 min bouts, %	20.1 ± 11.4	16.2 ± 7.5	−3.9 ± 12.0	0.402	0.069
**Wear time**					
Total waking wear min/day	869.4 ± 53.8	894.0 ± 35.4	24.7 ± 43.6	–	0.002
Total wear days	6.7 ± 0.7	6.7 ± 0.9	0.0 ± 1.2	–	0.884

Data presented as mean ± SD. Percentages reflect the proportion of total waking wear time

*P*-value calculated using paired t-tests

**Fig. 2:**
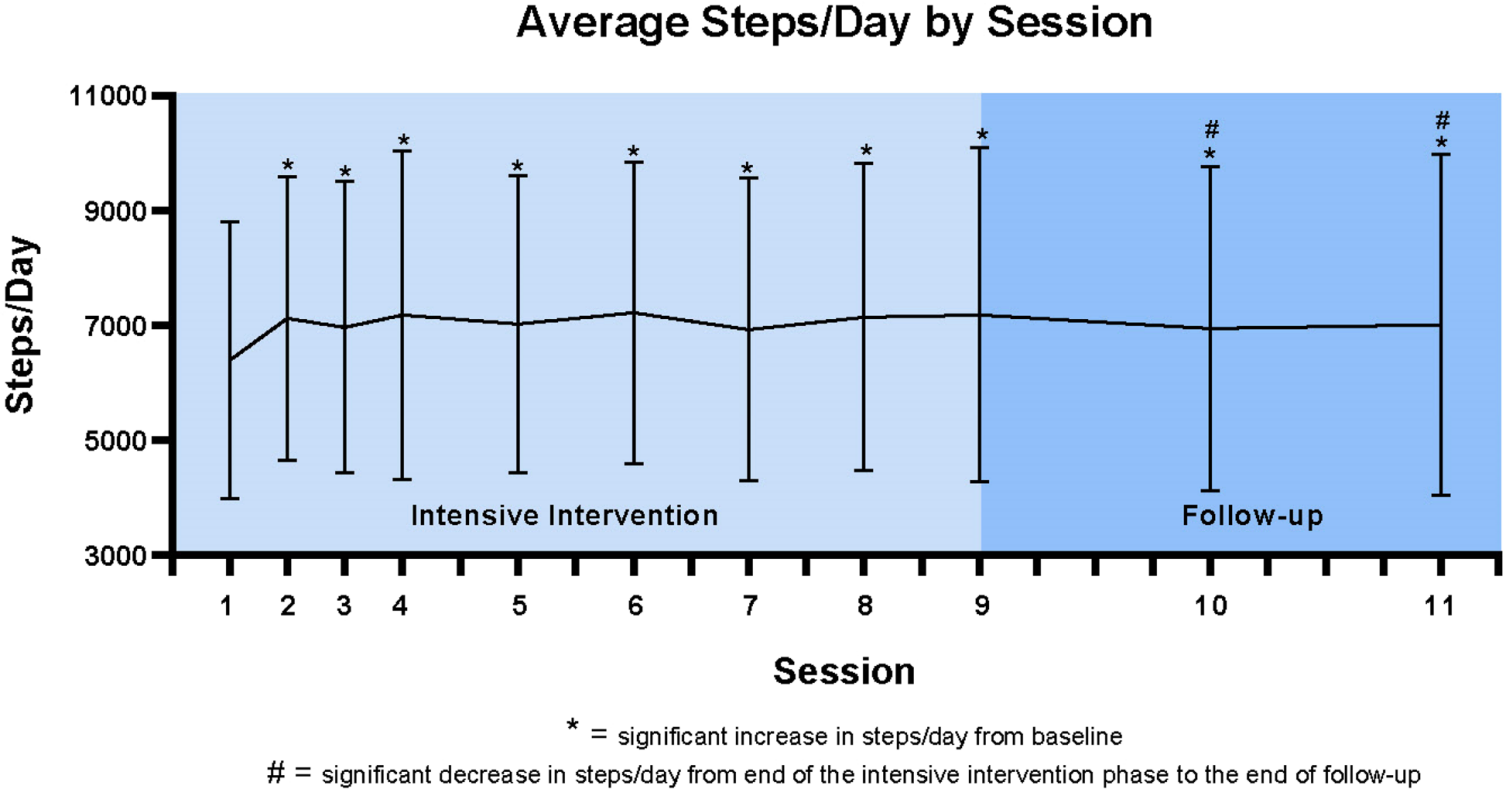
Changes in Fitbit Steps/Day by Health Coaching Session

As seen in Fig. 2, compared to Fitbit estimated steps at the beginning of the intervention (6389 ± 2409 steps/day), steps/day was significantly higher at each subsequent health coaching session through the end of the intensive intervention phase (7137 ± 2672steps/day), *p* < 0.01 for all comparisons. Compared to Fitbit estimated steps at the beginning of the follow-up phase (7178 ± 2910 steps/day), average steps/day was significantly lower for each subsequent health coaching session through the end of the follow-up phase (7007 ± 2974 steps/day), while remaining higher than baseline values, *p* < 0.02 for all comparisons. Across the intensive intervention phase, average step count increased by 749 steps/day. If these steps were accumulated at a rate of 100 steps per minute (corresponding to brisk walking and moderate intensity physical activity) [35], this would translate to 7.5 extra minutes of moderate intensity physical activity per day and approximately 50 additional minutes of moderate intensity physical activity per week.

## Discussion

The findings from the INSPiRE study provide evidence that a remotely-delivered health coaching intervention, paired with a wearable activity monitor, is feasible, highly acceptable, and effective at improving movement behaviors during pregnancy. All three objectives to determine program feasibility were met, including over 50% recruitment and enrollment of eligible participants who completed a screening form (71% recruitment and enrollment observed), greater than 85% retention (97% retention observed), attendance at 75% or more health coaching sessions (99% adherence observed), and syncing of Fitbit device on 75% or more days (99% adherence observed). For acceptability, 100% of participants reported that they were very satisfied or satisfied with the program. For preliminary efficacy, both activPAL and Fitbit data illustrated significant improvements in steps over the duration of the program, with the activPAL also illustrating significant decreases in sedentary time.

Two other pilot studies have examined the feasibility, acceptability, and efficacy of a remotely-delivered physical activity intervention including a wearable activity monitor in pregnant populations [17, 32]. Choi and colleagues conducted a 12-week randomized controlled trial where 30 pregnant women between 10 and 20 weeks gestation were assigned to an intervention (mobile phone app plus Fitbit) or control (Fitbit only) condition [32]. Enrollment rates for this study were similar to the INSPiRE study, with 64% of eligible women who completed a screening form randomized into the program. Adherence in Choi’s study was based on response rates for daily messages and completion of an activity diary. Adherence gradually decreased from approximately 80% in week 1 to 35% in week 12, lower than observed in the INSPiRE study. For efficacy, they observed an increase of + 1096 steps/day in the intervention group across the 12 weeks, compared to an increase of + 259 steps/day in control participants; however, the change between groups was not significantly different. The increase in steps/day observed in the intervention group was similar but smaller in magnitude than observed in the INSPiRE study over the intensive intervention phase (+ 1716 steps/day). This difference could in part be attributed to the differences in the approach used for intervention delivery. Choi et al., delivered the intervention primarily through a mobile health app using text messages, while the INSPiRE study intervention was delivered by a health coach via Zoom or telephone.

Larsen and colleagues also conducted a 12-week single-arm pilot trial where 17 pregnant women between 10 and 27 weeks gestation with diabetes were recruited to take part in a one-on-one counseling and goal-setting session and were provided with a Fitbit device for self-monitoring [17]. In this study, enrollment rates were slightly lower than the INSPiRE study, with approximately 40% of those eligible completing the baseline assessment. Of those who began the intervention, 76% completed a follow-up visit and adherence to Fitbit wear ranged from 27 to 100% of days (median wear time of 90%), indicating good adherence. Nearly all participants who completed the follow-up visits (12/13) indicated they were quite or extremely satisfied with the program. However, the intervention had a limited effect on steps. Mean daily steps increased from baseline through week three (+ 147 steps/day), but then decreased by week 12 (− 2078 steps/day). The differences observed in preliminary efficacy between Larsen et al. and the INSPiRE study may be attributed to differences in intervention delivery method, number of contacts (2 vs. 12), and study populations (women with vs. without diabetes).

Evidence on the preliminary efficacy of the INSPiRE study is promising for both increasing physical activity and decreasing sedentary behavior; however, interpretation is limited due to the lack of a control group for comparison. Notably, in non-pregnant populations an increase of ~ 1000 steps/day is associated with lower risk of all-cause mortality and CVD morbidity and mortality, and these health benefits are observed below 10,000 steps/day [36]. Our team has also shown that women with very low step counts across pregnancy trimesters (~ 5000 steps/day) are at a greater risk for adverse pregnancy outcomes, including hypertensive disorders of pregnancy, compared to those achieving 7800 steps/day or more [7]. It is possible, therefore, that the observed increase of + 1716 steps/day in INSPiRE participants could result in improved health outcomes and contribute to lower risk of adverse outcomes during pregnancy. In our sample of 34 participants, we had lower or similar rates of adverse pregnancy outcomes compared to rates observed generally among pregnant populations in the United States. For example, 3% of our participants experienced preterm delivery, compared to a national average of 10% [37]. Between 6 and 9% of our sample experienced intrauterine growth restriction, gestational hypertension, or gestational diabetes, which is consistent or lower than national estimates [37–39]. Compared to national averages, the percent of participants meeting gestational weight gain guidelines was higher (41.4% vs. 32.0%) [40], and fewer women had cesarean sections (11.8% vs. 31.8%) [41]. Offspring birth weight, BMIz, and Apgar scores were all within healthy ranges. At a minimum, the INSPiRE program does not appear to increase risk of adverse pregnancy outcomes for participants.

Our hypotheses that the intervention would increase physical activity levels and reduce sedentary behavior across the intensive intervention phase (second trimester) were largely supported. However, we did observe declines in physical activity (assessed via Fitbit) during the follow-up phase which occurred in the third trimester. The decline in physical activity in the third trimester of pregnancy is well documented [7, 42, 43]. While we were not able to prevent this decline from occurring, it is important to note that activity remained above baseline levels throughout the entire duration of the intervention, including the follow-up phase. Without a control group, it is difficult to draw conclusions, but it is possible that the intervention reduced the magnitude of decline typically observed in the third trimester of pregnancy.

The reductions observed for total sedentary time (− 4.2% of waking wear time; − 37.0 min/day) as well as sedentary time in bouts of 30 (− 4.1%; − 36.1 min/day) and 60 minutes (− 3.9%; − 34.4 min/day) is larger in magnitude than other sedentary behavior interventions delivered in non-pregnant populations. A meta-analysis of sedentary behavior interventions found an average reduction of 22 minutes/day in sedentary time in favor of the intervention vs. control groups [44]. Additional research is needed to better clarify how reductions in sedentary time relate to clinical health outcome measures in pregnant populations.

A notable strength of this study was the high adherence and compliance to the intervention protocol. Further, this study included both commercial (Fitbit) and research-grade (activPAL) assessment of physical activity and sedentary behavior, while prior studies are limited to commercial devices that are unable to capture some additional aspects of physical activity (e.g., standing time) and sedentary behavior. An additional strength of the INSPiRE program is that the intervention required no in-person contacts, and thus may be more appropriate for hard-to-reach populations, including those residing in rural or underserved areas. However, despite these strengths, this study also had several limitations. Most importantly, the single-arm design without a control group limits our ability to determine the efficacy of the intervention. Given the promising findings, the next logical step is to repeat this intervention study using a randomized controlled trial study design. The participants wore an activPAL monitor at baseline and at the end of the intensive intervention phase, but due to limited resources we were not able to repeat the activPAL wear protocol at the end of the follow-up phase. While we did have Fitbit data available in this phase, we were unable to examine changes in sedentary behavior in late pregnancy. Study findings also have limited generalizability as this was a largely homogenous population of highly educated and majority white women. Finally, the study population was relatively active at baseline, which may have limited the efficacy of the intervention to increase physical activity due to ceiling effects.

## Conclusions

The INSPiRE study demonstrated good feasibility, high acceptability, and preliminary efficacy for improving movement behaviors in women during pregnancy. Further, this study addresses limitations of previous interventions which largely employed structured, higher intensity, in-person supervised exercise. These preliminary findings are a promising first step toward identifying effective, remotely-delivered interventions to optimize movement behaviors in pregnancy and, in turn, improve the health of mothers and their babies. These data warrant future evaluation in a fully powered randomized controlled trial to further explore the intervention effects on physical activity and sedentary behavior during pregnancy, identify the most effective intervention components, and examine effects on maternal-child health outcomes.

## Data Availability

The datasets generated and/or analyzed during the current study are available in the University of Iowa repository, [10.25820/data.006170].

## Abbreviations

ACOG: American College of Obstetrics and Gynecology
MVPA: moderate-to-vigorous intensity physical activity
